# Associations Between Fatty Acid Levels in Human Blood and Trigeminovascular Tissues

**DOI:** 10.1002/lipd.70010

**Published:** 2025-09-21

**Authors:** Daisy Zamora, Mark S. Horowitz, Sharon F. Majchrzak‐Hong, Katherine Ness Shipley, Nicholas M. Salem, Ann I. Scher, Matthew R. Sapio, Michael J. Iadarola, Christopher E. Ramsden

**Affiliations:** ^1^ Lipid Peroxidation Unit, Laboratory of Clinical Investigation National Institute on Aging Baltimore Maryland USA; ^2^ Department of Physical Medicine and Rehabilitation University of North Carolina School of Medicine Chapel Hill North Carolina USA; ^3^ Intramural Program of the National Institute on Alcohol Abuse and Alcoholism NIH Bethesda Maryland USA; ^4^ Uniformed Services University of the Health Sciences Bethesda Maryland USA; ^5^ Department of Perioperative Medicine, Clinical Center National Institutes of Health Bethesda Maryland USA

**Keywords:** fatty acids, headache, meninges, migraine, oxylipins, trigeminal nerve

## Abstract

Omega‐3 and omega‐6 polyunsaturated fatty acids (PUFAs) are precursors to oxylipins that modulate pain and inflammation. We previously demonstrated that (1) a dietary intervention increasing omega‐3 and reducing omega‐6 PUFAs alters the concentration of these oxylipin precursors in blood, and (2) these changes are associated with reduced headache pain in humans. However, the extent to which blood levels reflect trigeminovascular tissues remains unclear. We sought to determine whether oxylipin precursor PUFA levels in blood reflect those in the meninges, cranial arteries, and trigeminal ganglia. Precursor PUFA compositions of post‐mortem blood and trigeminovascular tissue specimens from 70 individuals, procured from the Human Brain Collection Core at the National Institute of Mental Health, were quantified. Regression models adjusted for confounders examined relationships between blood and tissue PUFA levels. Eicosapentaenoic acid in blood was associated with levels in cranial arteries, meninges, and trigeminal ganglia [*logged coefficients* (*p* value): 0.29 (0.019); 0.37 (< 0.001); 0.25 (0.009)]. Other PUFAs, including linoleic acid, arachidonic acid, n‐6 docosapentaenoic acid, and docosahexaenoic acid, also showed significant associations between blood and meninges and/or trigeminal ganglia levels. These findings support using blood measurements of certain PUFAs as a proxy for their concentration in tissues directly involved in headache pathogenesis.

## 1 Introduction

**FIGURE 1:**
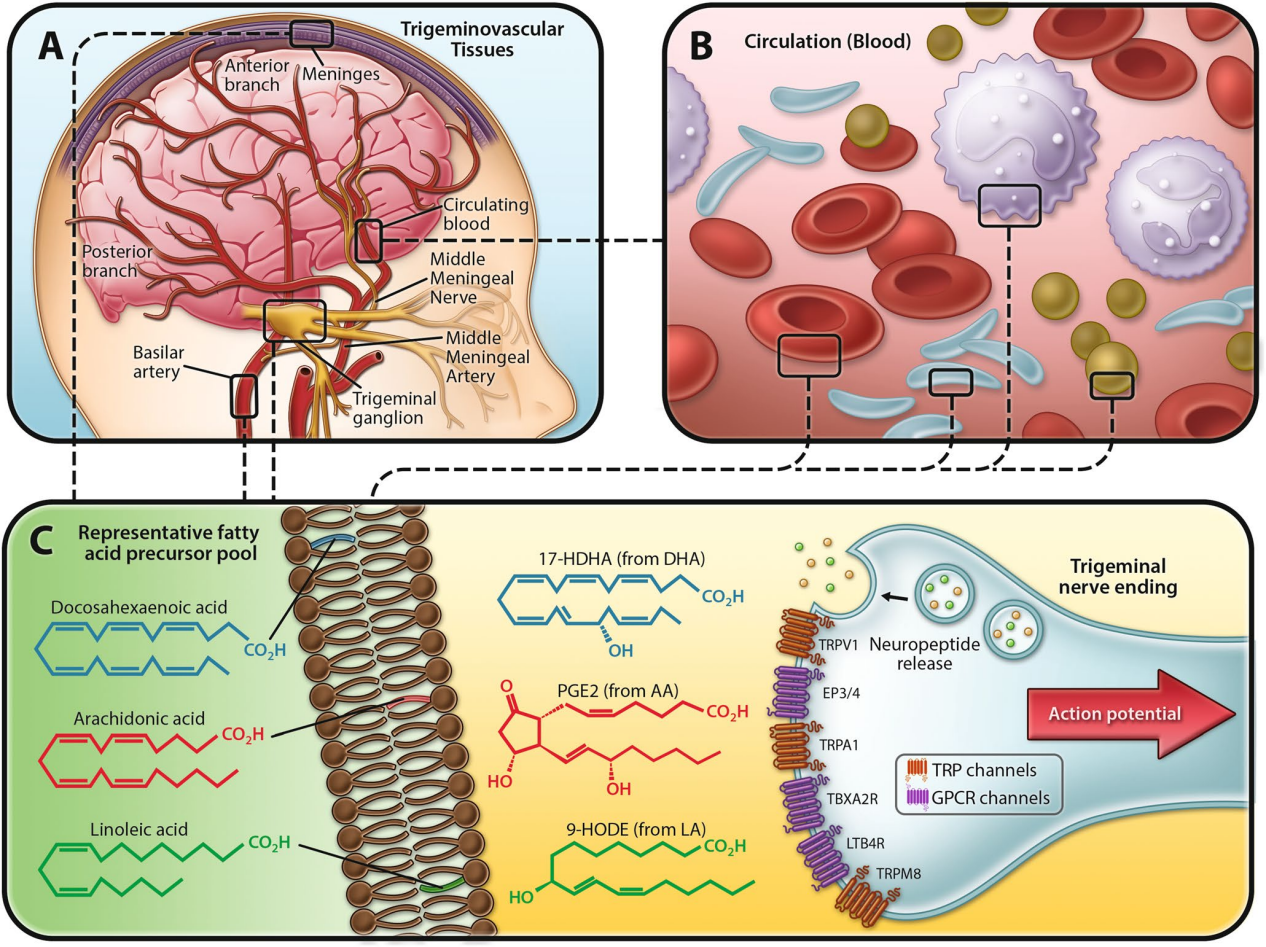
Rationale and biosynthetic pathways linking precursor omega‐6 and omega‐3 fatty acids and their oxylipin derivatives to headache pathogenesis. (A) Omega‐3 and omega‐6 fatty acids are major components of lipid membranes of tissues innervated by the trigeminal nerve including meninges, cranial vessels, mucosal membranes, the trigeminal nerve itself, and the lipid pools in pain processing pathways (i.e., myelin, astrocytes, and CNS neurons). (B) Circulating blood contains additional pools of precursor omega‐3 and omega‐6 fatty acids including immune cells, platelets, erythrocytes, and lipoproteins. (C) Collectively, these lipid pools serve as precursors for synthesis of bioactive oxylipins with pro‐ and antinociceptive properties including prostaglandins, leukotrienes, octadecanoids, resolvins, and omega‐3 monoepoxides. Oxylipins activate GPCRs and TRP channel receptors to regulate trigeminal nerve activation and stimulate release of headache‐related neuropeptides (e.g., CGRP). Six oxylipin receptors that are enriched in human trigeminal nerve are shown. Abbreviations: LA, linoleic acid; AA, arachidonic acid; DHA, docosahexaenoic acid; HDHA, hydroxydocosahexaenoic acid; TRP, transient receptor potential vanilloid; GPCR, G‐protein coupled receptor; EP, E‐prostanoid; TBX, thromboxane; LT, leukotriene; CGRP, calcitonin gene related peptide.

Omega‐3 and omega‐6 polyunsaturated fatty acids (PUFAs), which are major components of lipid membranes in human tissues, serve as precursors to oxylipins that modulate pain and inflammation and are implicated in headache pathogenesis (Ramsden et al. 2021). In preclinical models, oxylipins derived from omega‐3 eicosapentaenoic acid (EPA) and docosahexaenoic acid (DHA) exhibit anti‐nociceptive and anti‐inflammatory properties (Dyall et al. 2022). In contrast, oxylipins derived from omega‐6 arachidonic acid (AA), such as prostaglandins and leukotrienes, have well‐established pro‐nociceptive, pro‐inflammatory, and vasodilatory properties (Dyall et al. 2022). Emerging preclinical and clinical evidence also implicates oxylipin derivatives of omega‐6 linoleic acid (LA) in pain signaling (Patwardhan et al. 2009; Boyd et al. 2021; Doolen et al. 2020; Domenichiello et al. 2020; Ramsden et al. 2017; Mann et al. 2018) and headache pathogenesis (Ramsden et al. 2021; Doolen et al. 2020; Domenichiello et al. 2020). Several oxylipin receptors are reported to be highly expressed in human trigeminal ganglia (Figure 1) (LaPaglia et al. 2018). Moreover, certain oxylipins have been shown to modulate the release of the headache‐related neuropeptide, calcitonin gene‐related peptide (CGRP), through the regulation of transient receptor potential (TRP) channel activation (Sisignano et al. 2016; Ciardo and Ferrer‐Montiel 2017; Zimmer et al. 2018; Iannone et al. 2022), providing a potential mechanism linking oxylipins and their precursor PUFAs to the initiation and perpetuation of headaches.

We have previously demonstrated that a controlled dietary intervention increasing omega‐3 and reducing omega‐6 PUFA intake can reduce the frequency and severity of headache pain (Ramsden et al. 2021, 2013; MacIntosh et al. 2021; Ramsden, Faurot, et al. 2015; Ramsden, Zamora, et al. 2015). This intervention altered oxylipin precursor PUFAs in circulating lipid pools, including plasma, erythrocytes, and immune cells (Ramsden et al. 2021; Domenichiello et al. 2020; Ramsden et al. 2013, 2012), supporting biological plausibility (Figure 1). We also found that diet‐induced changes in several PUFAs (e.g., an increase in circulating EPA and DHA) were associated with reduced headache pain (Ramsden et al. 2021, 2013). However, the abundance of specific precursor PUFAs in human trigeminovascular tissues is not yet known and the extent to which blood PUFA levels reflect tissues directly implicated in headache pathogenesis remains unclear.

The trigeminovascular system—which consists of the trigeminal nerve and its innervated tissues such as the meninges and cranial vessels—plays a central role in the pathogenesis of primary headaches, including migraine (Burstein et al. 2015; Eftekhari et al. 2015). Trigeminal neurons are primarily responsible for conveying nociceptive signals from innervated tissues to the brain, a process central to the headache phase of migraine (Burstein et al. 2015; Eftekhari et al. 2015). Meninges are a major source of nociceptive oxylipins such as PGE2 (Billotte and Vesin 1997), which are implicated in migraine pathogenesis. Cranial vessels, such as basilar and middle meningeal arteries, are implicated in migraine pathogenesis, in part by releasing vasoactive and nociceptive mediators, including CGRP (Brennan and Charles 2010).

For this study, we used human donor post‐mortem trigeminovascular tissues (meninges, basilar arteries, and trigeminal ganglia), blood, and medical records. The main goals were to characterize the precursor PUFA composition of tissues that are implicated in headache pathogenesis and to determine whether blood is a proxy for the PUFA levels of these tissues. In addition, we sought to explore whether tissue donors with a history of headache or migraine had lower amounts of omega‐3 and higher omega‐6 in these tissues relative to individuals with no history of headache or migraine.

## 2 Materials and Methods

### 2.1 Tissue Procurement and Cohort Selection

The tissues for this study were obtained from the Human Brain Collection Core (HBCC) at the National Institute of Mental Health (NIMH). Tissues are procured through methods approved by the HBCC Oversight Committee and the National Institutes of Health (NIH) Department of Bioethics and with the permission of the families (National Institute of Mental Health, n.d.). HBCC's methods have been previously described in detail (Lipska et al. 2006). This repository includes tissue and blood samples from individuals who had various psychiatric conditions and those who died by suicide as well as samples from individuals without psychiatric diagnoses. Medical chart review was performed in collaboration with the HBCC to identify tissue donors with and without a documented history of headache or migraine. Individuals with any mention of headache or migraine in medical charts were considered to have a documented history of headaches and categorized as headache “cases.” Individuals with no mention of headache or migraine in medical charts were classified as non‐headache “controls.” However, whereas some tissue donors had extensive medical records, others had almost none, making it difficult to accurately determine the frequency/severity of headache as well as properly characterize tissue donors who did not experience headache. The headache and non‐headache groups were frequency‐matched based on sex and three strata of age at death.

### 2.2 Tissue Collection, Processing, and Fatty Acid Analysis

We analyzed specimens from 70 subjects with whole blood and at least one of the three target tissues. Specimens were transported on dry ice and stored at −80°C until analysis. Tissues were homogenized and processed using a modified Bligh and Dyer extraction method. Omega‐3 and omega‐6 fatty acids of interest were extracted and analyzed by gas chromatography with a flame ionization detector as previously described (Mann et al. 2018; MacIntosh et al. 2021), in the Laboratory of Membrane Biochemistry and Biophysics in the Intramural Program of the National Institute on Alcohol Abuse and Alcoholism (NIAAA). Briefly, fatty acids were extracted from samples by using a modification of the method of Folch et al. (1957) by using chloroform/methanol solvents in a 1:1 ratio and an aqueous phosphate buffer solution. Lipids were recovered into the chloroform layer after two extractions. This fraction was evaporated under nitrogen and transesterified. Fast gas chromatography was performed on the methyl esters in hexane with a Hewlett‐Packard 6890 gas chromatograph equipped with a flame ionization detector, as previously described (Ramsden et al. 2013, 2016). Peaks were identified by using authentic standards (Nu‐Check Prep, Elysian, MN). Fatty acid concentrations in blood were quantified by comparison with peak areas of the identified 22:3n‐3 internal standard peaks. Fatty acids are presented as a percent of total fatty acids as a normalization technique. We selectively evaluated the following PUFAs based on their role as oxylipin precursors: alpha‐linolenic acid (ALA, C18:3n‐3), EPA (C20:5n‐3), DHA (C22:6n‐3), docosapentaenoic acid (DPA n‐3, C22:5n‐3), LA (C18:2n‐6), AA (C20:4n‐6), dihomo‐gamma‐linolenic acid (DGLA, C20:3n‐6), docosatetraenoic acid (DTA, C22:4n‐6), and docosapentaenoic acid (DPA n‐6, C22:5n‐6).

### 2.3 Data Analysis

No statistical methods were used to pre‐determine sample sizes. When using non‐normally distributed variables, we used either non‐parametric statistics or log transformations. Descriptive statistics were used to quantify the abundance of each targeted fatty acid in blood, meninges, basilar arteries, and trigeminal ganglia. The associations between PUFAs in circulation and those in trigeminovascular tissues were assessed using Spearman's correlations as well as linear regression models of log‐transformed fatty acids adjusted for headache status, age, body mass index (BMI), sex, race, manner of death, post‐mortem interval (PMI), geographic source, and positive ethyl alcohol test. Wilcoxon rank‐sum tests were used to compare the abundance of each fatty acid in those with and without a documented history of headache. Statistical analyses were performed using Stata version 18.5 with a significance threshold set at *p* < 0.05 using two‐sided tests.

To evaluate whether the length of the PMI modified the association between blood and tissue fatty acid concentrations, we included an interaction term between blood fatty acid levels and PMI (continuous, hours) in the adjusted regression models. A significant interaction coefficient indicates that the strength of the blood–tissue association varies across the range of PMIs.

## 3 Results

### 3.1 Demographics

Demographic and clinical characteristics of the cohort are provided in Table 1. The cohort had a mean age of 41 years, and it was 54% female and 77% white. Just over half of the subjects attended college or had post‐graduate studies, and 83% had a psychiatric diagnosis. Mean PMI was 32 h with a range of 12–64 h. The headache and non‐headache groups were comparable in most measured characteristics except race and geographic source, where a larger proportion of donors with documented headaches were white and living in Virginia.

**TABLE 1:** Demographic and clinical characteristics of postmortem cohort.

	Control (*n* = 35)	Headache (*n* = 35)	Total (*n* = 70)	*p* ^a^
Demographic characteristics				
Age at death, years, mean (SD)	39.8 (13.3)	42.4 (14.1)	41.1 (13.7)	0.421
Body mass index, mean (SD)	31.2 (11.5)	30.5 (7.2)	30.9 (9.6)	0.758
Female, *n* (%)	19 (54%)	19 (54%)	38 (54%)	1.000
Race, *n* (%)				
Black	10 (29%)	2 (6%)	12 (17%)	0.002
White	21 (60%)	33 (94%)	54 (77%)	
Other	4 (11%)	0 (0%)	4 (6%)	
Relationship status, *n* (%)				
Single	19 (54%)	13 (37%)	32 (46%)	0.190
Partnered	8 (23%)	15 (43%)	23 (33%)	
Divorced, separated, or widowed	8 (23%)	7 (20%)	15 (21%)	
Highest level of education completed, *n* (%)				
≤ High school diploma	18 (53%)	15 (43%)	33 (48%)	0.701
Some college, associate's, or bachelor's	11 (32%)	14 (40%)	25 (36%)	
Post‐graduate	5 (15%)	6 (17%)	11 (16%)	
Clinical characteristics				
Manner of death, *n* (%)				
Natural	15 (43%)	7 (22%)	22 (33%)	0.132
Suicide	12 (34%)	18 (56%)	30 (45%)	
Other	8 (23%)	7 (22%)	15 (22%)	
Post‐mortem interval, hours, mean (SD)	34.0 (12.4)	29.5 (12.5)	31.9 (12.5)	0.144
Source, *n* (%)				
Virginia	22 (63%)	33 (94%)	55 (79%)	0.001
Washington, D.C.	13 (37%)	2 (6%)	15 (21%)	
NIMH primary psychiatric diagnosis, *n* (%)				
No psychiatric diagnosis	7 (21%)	4 (13%)	11 (17%)	0.355
Bipolar depression	7 (21%)	6 (20%)	13 (20%)	
Unipolar depression	12 (35%)	17 (57%)	29 (45%)	
Schizophrenia	3 (9%)	0 (0%)	3 (5%)	
Substance use disorder	2 (6%)	2 (7%)	4 (6%)	
Other	3 (9%)	1 (3%)	4 (6%)	
ETOH, positive result, *n* (%)	9 (26%)	7 (21%)	16 (24%)	0.662
*Pain* ^b^				
Non‐headache chronic (verified), *n* (%)	9 (26%)	22 (63%)	31 (44%)	0.002
Back, *n* (%)	3 (9%)	12 (34%)	15 (21%)	0.009
Neck, *n* (%)	0 (0%)	6 (17%)	6 (9%)	0.010
Fibromyalgia/myocardial, *n* (%)	0 (0%)	3 (9%)	3 (4%)	0.077
Gastrointestinal, *n* (%)	2 (6%)	6 (17%)	8 (11%)	0.133
Other, *n* (%)	7 (20%)	10 (29%)	17 (24%)	0.403
Any, *n* (%)	9 (26%)	22 (63%)	31 (44%)	0.002
Smoker, *n* (%)	17 (49%)	17 (49%)	34 (49%)	1.000
*Substance abuse* ^b^				
Alcohol, *n* (%)	13 (39%)	15 (43%)	28 (41%)	0.772
Cocaine, *n* (%)	5 (15%)	5 (14%)	10 (15%)	0.920
Opioid, *n* (%)	11 (33%)	11 (31%)	22 (32%)	0.867
Polysubstance, *n* (%)	7 (21%)	7 (20%)	14 (21%)	0.902
Cannabis, *n* (%)	3 (9%)	5 (14%)	8 (12%)	0.506
Any, *n* (%)	16 (48%)	21 (60%)	37 (54%)	0.341

Abbreviations: *n* = count; NIMH = National Institute of Mental Health; SD = standard deviation.

^a^ Between‐group *p* values were determined with t‐tests for continuous variables and Pearson's chi‐squared test for categorical variables.

^b^ Subjects can have multiple categories.

### 3.2 Tissue Fatty Acid Composition

**FIGURE 2:**
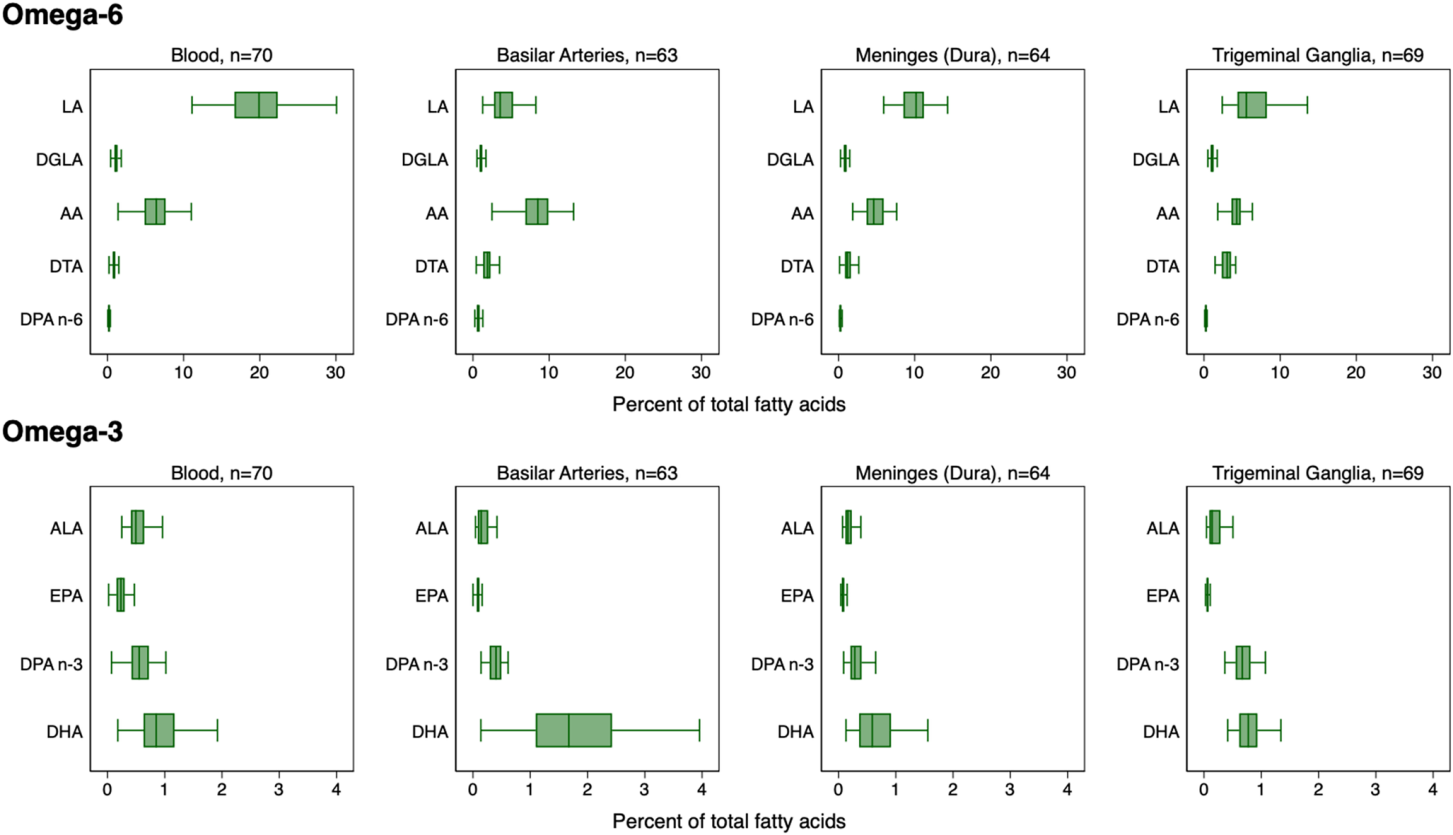
Fatty acid profiles by tissue. These box plots show the distributions of fatty acids in each tissue as a percent of total fatty acids. Note that the *x*‐axis scale in the first row is 0%–30%, and the *x*‐axis scale in the second row is 0%–4%. Outside values are not shown.

Figure 2 provides a comparative profile of PUFA levels in blood, basilar arteries, meninges, and trigeminal ganglia. LA was the most abundant omega‐6 PUFA in blood, meninges, and trigeminal ganglia, while AA was the most abundant in basilar arteries. DGLA, DTA, DPA n‐6, ALA, EPA, DPA n‐3, and DHA were comparatively low in all tissues. Medians and interquartile ranges for these and other fatty acids (a total of 24) are listed in Table S1.

### 3.3 Associations Between Fatty Acids in Blood and Trigeminovascular Tissue

In Table 2, associations between PUFA concentrations in blood and each of the three trigeminovascular tissues were evaluated using Spearman correlations and adjusted regressions. For omega‐6 PUFAs, blood LA showed no statistically significant correlations with basilar arteries, meninges, or trigeminal ganglia based on Spearman's *rho* (all *p* > 0.05). However, adjusted regression analysis indicated a strong positive association between LA levels in blood and meninges (*coefficient* = 0.49; 95% CI, 0.28–0.71; *p* < 0.001). DGLA displayed a significant correlation in trigeminal ganglia (*rho* = 0.25, *p* = 0.035) in the Spearman analysis and achieved statistical significance in the regression model for both basilar arteries (*coefficient* = 0.23; 95% CI, 0.03–0.42; *p* = 0.023) and trigeminal ganglia (*coefficient* = 0.33; 95% CI, 0.16–0.49; *p* < 0.001). In contrast, AA demonstrated no significant Spearman correlation in any tissue (all *p* > 0.05), yet it was significantly associated with AA levels in trigeminal ganglia in the regression model (*coefficient* = 0.27; 95% CI, 0.08–0.46; *p* = 0.005).

**TABLE 2:** Association between blood and trigeminovascular tissue fatty acids.

	Basilar arteries		Meninges (Dura)		Trigeminal ganglia	
	Rho	*p*	Rho	*p*	Rho	*p*
Spearman's correlations^a^						
	*n* = 63		*n* = 64		*n* = 69	
Omega‐6						
LA	0.19	0.136	0.17	0.173	0.12	0.321
DGLA	0.25	0.052	0.14	0.256	0.25	**0.035**
AA	−0.11	0.396	0.17	0.170	−0.01	0.906
DTA	−0.07	0.602	0.05	0.719	0.19	0.123
DPA n‐6	0.08	0.520	0.33	**0.009**	0.47	**< 0.001**
Omega‐3						
ALA	0.03	0.810	0.41	**< 0.001**	0.19	0.119
EPA	0.37	**0.004**	0.52	**< 0.001**	0.39	**0.001**
DPA n‐3	0.02	0.847	0.28	**0.025**	0.46	**< 0.001**
DHA	−0.01	0.957	0.25	**0.043**	0.30	**0.014**

	Coefficient(95% CI)	*p*	Coefficient(95% CI)	*p*	Coefficient(95% CI)	*p*
Adjusted regressions^b^						
	*n = 57*		*n = 58*		*n = 63*	
Omega‐6						
LA	0.51 (−0.12, 1.13)	0.112	0.49 (0.28, 0.71)	**< 0.001**	0.30 (−0.16, 0.76)	0.196
DGLA	0.23 (0.03, 0.42)	**0.023**	0.19 (−0.03, 0.41)	0.087	0.33 (0.16, 0.49)	**< 0.001**
AA	0.07 (−0.30, 0.45)	0.689	0.06 (−0.18, 0.30)	0.613	0.27 (0.08, 0.46)	**0.005**
DTA	−0.09 (−0.35, 0.18)	0.511	−0.12 (−0.34, 0.10)	0.280	0.15 (0.02, 0.28)	**0.021**
DPA n‐6	0.13 (−0.19, 0.44)	0.420	0.35 (0.13, 0.57)	**0.002**	0.50 (0.33, 0.67)	**< 0.001**
Omega‐3						
ALA	0.34 (−0.08, 0.77)	0.114	0.64 (0.38, 0.90)	**< 0.001**	0.36 (0.02, 0.70)	**0.041**
EPA	0.43 (0.03, 0.83)	**0.035**	0.42 (0.26, 0.58)	**< 0.001**	0.24 (0.05, 0.43)	**0.016**
DPA n‐3	−0.05 (−0.28, 0.17)	0.636	0.22 (0.02, 0.42)	**0.034**	0.29 (0.17, 0.41)	**< 0.001**
DHA	0.04 (−0.48, 0.55)	0.891	0.13 (−0.23, 0.48)	0.480	0.32 (0.15, 0.48)	**< 0.001**

*Note: p* values in bold are less than 0.05.

Abbreviation: CI = confidence interval.

^a^ Each of the 70 cases had a blood sample and at least one tissue sample.

^b^ Regression analyses were conducted with the fatty acids (as percent of total fatty acids) transformed to natural logarithms to stabilize variance and improve model fit. Coefficients reflect the percent change in tissue fatty acid associated with a 1% increase in blood fatty acid. Regressions were adjusted for the following potential confounders: headache status, age, body mass index, sex, race, manner of death, postmortem interval, geographic source, and positive ethyl alcohol test. Differences in sample sizes from Spearman's correlations are due to missing confounders.

DPA n‐6 exhibited significant correlations with both the meninges (*rho* = 0.33, *p* = 0.009) and the trigeminal ganglia (*rho* = 0.47, *p* < 0.001), with adjusted regression models also showing significant associations in the meninges (*coefficient* = 0.35; 95% CI, 0.13–0.57; *p* = 0.002) and the trigeminal ganglia (*coefficient* = 0.50; 95% CI, 0.33–0.67; *p* < 0.001).

Among omega‐3 PUFAs, ALA displayed a significant positive correlation with tissue levels in the meninges in both Spearman's analysis (*rho* = 0.41, *p* < 0.001) and regression (*coefficient* = 0.64; 95% CI, 0.38–0.90; *p* < 0.001); and in trigeminal ganglia in the regression (*coefficient* = 0.36; 95% CI, 0.02–0.70; *p* = 0.041). EPA showed robust associations in all analyses, with significant correlations in basilar arteries (*rho* = 0.37, *p* = 0.004), meninges (*rho* = 0.52, *p* < 0.001), and trigeminal ganglia (*rho* = 0.41, *p* < 0.001). These correlations were further supported by adjusted regression models in basilar arteries (*coefficient* = 0.43; 95% CI, 0.03–0.83; *p* = 0.035), meninges (*coefficient* = 0.42; 95% CI, 0.26–0.58; *p* < 0.001), and trigeminal ganglia (*coefficient* = 0.24; 95% CI, 0.05–0.43; *p* = 0.016), demonstrating consistent positive associations between blood EPA levels and tissue concentrations in the headache‐implicated regions. DHA in blood was positively correlated with DHA levels in the meninges (*rho* = 0.25, *p* = 0.043) and trigeminal ganglia (*rho* = 0.30, *p* = 0.014). In the regression models, DHA had a significant association with trigeminal ganglia levels only (*coefficient* = 0.32; 95% CI, 0.15–0.48; *p* < 0.001). DPA n‐3 showed significant correlations in the meninges (*rho* = 0.28, *p* = 0.025) and trigeminal ganglia (*rho* = 0.46, *p* < 0.001), with regression results further confirming associations in the meninges (*coefficient* = 0.22; 95% CI, 0.02–0.42; *p* = 0.034) and trigeminal ganglia (*coefficient* = 0.29; 95% CI, 0.17–0.41; *p* < 0.001). These findings suggest that blood levels of certain omega‐3 and omega‐6 PUFAs, particularly EPA, DHA, ALA, and DPA (both omega‐3 and omega‐6 forms), could serve as proxies for tissue levels in headache pathogenesis, especially in tissues like the meninges and trigeminal ganglia.

Interaction analyses showed that for most fatty acids, blood–tissue associations were consistent across the range of PMI (Table S2). However, in the meninges, the interaction terms were significant for ALA (*coefficient* = 0.028; 95% CI, 0.003–0.053; *p* = 0.028) and DPA n‐3 (*coefficient* = −0.024; 95% CI, −0.046 to −0.003; *p* = 0.029), indicating that, with longer PMI, the association between blood and meningeal tissue strengthened for ALA and weakened for DPA n‐3.

### 3.4 Fatty Acid Composition According to Headache History

We used Wilcoxon rank‐sum tests to explore differences in PUFA composition of trigeminovascular tissues between individuals with and without a documented history of headaches. Table S3 shows the percent of total PUFA concentrations across blood, basilar arteries, meninges, and trigeminal ganglia stratified by headache history status. No statistical differences were observed in PUFA concentrations between headache cases versus non‐headache controls (all *p* > 0.05). We also conducted regression models (Table S4) to determine if headache status (independent variable) predicted PUFA composition (percent of total; logged), adjusting for age, BMI, sex, race, manner of death, PMI, geographic source, and positive ethyl alcohol test. There was no indication that PUFA composition differed between people with and without a headache history.

## 4 Discussion

This study sought to characterize the PUFA composition of trigeminovascular tissues implicated in headache pathogenesis and assess whether PUFA levels in blood might serve as proxies for levels in these tissues. Our findings revealed significant associations between certain omega‐3 and omega‐6 PUFAs in blood and their corresponding concentrations in the meninges and trigeminal ganglia, two tissues central to the pathophysiology of primary headaches. Notably, EPA, DHA, ALA, and both n‐3 and n‐6 forms of DPA showed strong correlations with tissue levels, suggesting that blood measurements of these PUFAs could be used as proxies of tissue PUFA composition in headache‐related studies. Across most fatty acids, we did not observe significant interaction between blood levels and PMI, indicating that the strength of blood–tissue associations was generally consistent regardless of PMI (Table S2). Notably, only meningeal ALA and DPA n‐3 showed modest PMI interactions, which may reflect tissue‐specific processes rather than generalized degradation.

The robust associations observed between omega‐3 PUFAs in blood and their concentration in the meninges and trigeminal ganglia support their potential as systemic markers of tissue‐level PUFA composition in headache‐relevant regions. These associations are noteworthy given the role of trigeminal ganglia in conveying nociceptive signals (Noseda and Burstein 2013; Yamanaka et al. 2021) as well as the meninges' role in releasing pro‐nociceptive and vasoactive mediators in headache pathophysiology (Brennan and Charles 2010). Omega‐6 PUFAs such as LA and AA showed less consistent associations with tissue levels, except for DPA n‐6, which demonstrated robust associations in both the meninges and trigeminal ganglia.

The study did not find significant differences in tissue PUFA composition between individuals with and without documented history of headaches. This lack of difference could be due to uniformly low omega‐3 PUFA intake within the studied population, as suggested by the notably lower omega‐3 PUFA blood levels compared to other US populations (Ramsden et al. 2021, 2013). Additionally, headache status based on medical record documentation may have limited sensitivity and specificity, as it is reliant on the available clinical documentation, which likely does not fully capture all headache cases or subtypes (i.e., some donor charts contained extensive documentation while others contained only a few pages).

We previously showed that the concentration of these PUFAs in blood is responsive to dietary changes (Ramsden et al. 2021, 2013). Our present findings of the association of blood PUFAs with trigeminovascular tissue levels suggest that changes in dietary PUFA intake may impact the oxylipin profiles within these critical pain‐related structures. In light of growing preclinical evidence implicating oxylipins derived from LA, AA, EPA, and DHA in nociceptive signaling (Ramsden et al. 2021; Dyall et al. 2022; Patwardhan et al. 2009; Boyd et al. 2021; Doolen et al. 2020; Domenichiello et al. 2020; Ramsden et al. 2017; Ciardo and Ferrer‐Montiel 2017; Zimmer et al. 2018; Iannone et al. 2022; Ramsden, Faurot, et al. 2015; Ramsden, Zamora, et al. 2015; Ramsden et al. 2012; Noseda and Burstein 2013; Antonova et al. 2012), our findings provide plausible mechanisms to explain the observed diet‐induced headache reductions. Combined with our previous research, these findings suggest that blood PUFA measurements can be used to monitor and potentially guide dietary interventions aimed at reducing headache pain. By increasing omega‐3 and reducing omega‐6 PUFA intake, it may be possible to favorably alter the PUFA composition in headache‐relevant tissues, thereby modulating the production of oxylipins involved in pain and inflammation.

This study has a few limitations. While we adjusted for several confounders, other unmeasured factors could influence PUFA levels in both blood and tissue. Moreover, the convenience sampling applied for this study can lead to type II errors (due to low power) and limits the generalizability of the findings. Last, the cohort was not specifically designed for headache research, and headache history was retrospectively determined from medical records, which could lead to misclassification of headache and non‐headache group assignment.

This study fills a key research gap by providing the precursor PUFA compositions of trigeminovascular tissues in humans and demonstrates that blood omega‐3 and omega‐6 PUFA levels reflect the composition of tissues directly implicated in headache pathogenesis. These findings support the use of blood PUFA measurements as proxies for tissue levels in dietary trials targeting headache conditions, offering a potential mechanism for dietary interventions to modulate physical pain.

## Author Contributions

Daisy Zamora refined the research questions, applied statistical methods, and substantially revised and expanded the manuscript. Mark S. Horowitz analyzed the data, prepared tables and figures, and coordinated the submission. Sharon F. Majchrzak‐Hong, Katherine Ness Shipley, Nicholas M. Salem, Ann I. Scher, Matthew R. Sapio, and Michael J. Iadarola carried out the research. All authors contributed to and approved the final version of the manuscript. Christopher E. Ramsden conceived and designed the study and wrote the first draft of the manuscript.

## Disclosure

The views, information or content, and conclusions presented do not necessarily represent the official position or policy of, nor should any official endorsement be inferred on the part of, the Clinical Center, the National Institutes of Health, or the Department of Health and Human Services.

## Conflicts of Interest

The authors declare no conflicts of interest.

## Supporting information


**Table S1:** Median (IQR) concentrations of fatty acids in various tissues.
**Table S2:** Effect of post‐mortem interval on association between blood and trigeminovascular tissue fatty acids.
**Table S3:** Percent of total fatty acids by headache group.
**Table S4:** Difference in percent of total fatty acids between headache status groups.

## Data Availability

The data that support the findings of this study are available from the corresponding author upon reasonable request.
