# Supplementary material for: Associations Between Fatty Acid Levels in Human Blood and Trigeminovascular Tissues
Source: Lipids. 2025 Sep 21;61(1):55–63. doi: 10.1002/lipd.70010 (PMC12780466; doi:10.1002/lipd.70010)
Supplement: Supplementary file 1 — Table S1: Median (IQR) concentrations of fatty acids in various tissues. Table S2: Effect of post‐mortem interval on association between blood and trigeminovascular tissue fatty acids. Table S3: Percent of total fatty acids by headache group. Table S4: Difference in percent of total fatty acids between headache status groups. [file LIPD-61-55-s001.pdf]

Supplement

Supplementary Table 1. Median (IQR) concentrations of fatty acids in various tissues

	Blood, n=64		Basilar Arteries, n=61	Meninges (Dura), n=64	Trigeminal Ganglia, n=63
	% of total FA	µg/mL	% of total FA	% of total FA	% of total FA
Omega-6 PUFAs					
LA	20.1 (16.8-22.3)	600 (453-753)	3.5 (2.8-5.1)	10.2 (8.5-11.3)	5.6 (4.4-8.4)
GLA	0.20 (0.13-0.28)	6.0 (4.0-9.0)	0.06 (0.04-0.11)	0.09 (0.07-0.11)	0.03 (0.02-0.04)
DGLA	1.1 (0.9-1.3)	31.5 (23.8-41.5)	1.0 (0.9-1.2)	0.9 (0.7-1.1)	1.1 (0.9-1.3)
AA	6.5 (5.0-7.7)	174 (146-213)	8.5 (6.9-9.9)	4.6 (3.7-5.9)	4.3 (3.6-4.8)
DTA	0.9 (0.7-1.1)	26.0 (20.0-32.0)	1.9 (1.4-2.3)	1.2 (0.9-1.6)	3.0 (2.3-3.6)
DPA n-6	0.21 (0.15-0.25)	6.0 (4.0-7.2)	0.64 (0.53-0.90)	0.25 (0.18-0.31)	0.24 (0.19-0.29)
Eicosadienoic acid	0.29 (0.26-0.33)	8.0 (7.0-10.0)	0.30 (0.26-0.35)	0.35 (0.29-0.42)	1.01 (0.79-1.20)
Omega-3 PUFAs					
ALA	0.48 (0.41-0.63)	16.0 (11.0-22.2)	0.14 (0.08-0.26)	0.16 (0.12-0.23)	0.14 (0.09-0.29)
EPA	0.24 (0.19-0.32)	7.0 (5.0-10.2)	0.09 (0.06-0.11)	0.07 (0.06-0.10)	0.06 (0.05-0.08)
DPA n-3	0.57 (0.42-0.74)	16.0 (12.8-21.0)	0.40 (0.29-0.50)	0.28 (0.21-0.40)	0.67 (0.54-0.81)
DHA	0.87 (0.63-1.20)	24.0 (18.8-33.5)	1.67 (1.13-2.43)	0.59 (0.36-0.92)	0.77 (0.61-0.95)
MUFAs					
Palmitoleic acid	1.7 (1.2-2.8)	45.5 (32.0-98.0)	0.6 (0.5-0.8)	1.0 (0.7-1.6)	0.9 (0.6-1.3)
Oleic acid	20.9 (18.3-23.2)	561 (447-904)	17.8 (15.7-19.7)	21.1 (19.5-23.8)	30.9 (29.3-32.8)
Cis-vaccenic acid	1.9 (1.7-2.1)	55.0 (38.8-78.2)	3.0 (2.4-3.6)	2.0 (1.8-2.3)	2.7 (2.5-2.9)
Eicosenoic acid	0.2 (0.2-0.3)	6.0 (5.0-9.2)	0.8 (0.4-1.4)	0.5 (0.4-0.6)	2.9 (2.5-3.2)
Erucic acid	0.06 (0.05-0.07)	2.0 (1.0-2.0)	0.29 (0.24-0.42)	0.49 (0.37-0.73)	0.50 (0.44-0.57)
Nervonic acid	1.7 (1.2-2.3)	49.5 (40.8-56.2)	2.5 (2.1-3.4)	1.8 (1.5-2.0)	4.8 (3.5-5.7)
SFAs					
Lauric acid	0.13 (0.08-0.18)	4.0 (2.0-7.0)		0.18 (0.12-0.22)	
Myristic acid	1.2 (0.9-1.4)	33.0 (21.2-50.2)	1.0 (0.9-1.1)	0.9 (0.8-1.2)	1.5 (1.3-1.8)
Palmitic acid	26.9 (25.1-28.1)	752 (628-987)	25.7 (23.0-27.2)	31.1 (29.1-33.0)	18.8 (17.1-20.6)
Stearic acid	9.7 (8.5-10.4)	280 (227-339)	21.2 (19.9-22.8)	17.3 (15.4-19.0)	10.2 (9.2-11.3)
Arachidic acid (20:0)	0.31 (0.26-0.37)	9.0 (7.0-11.0)	0.69 (0.59-0.88)	0.76 (0.67-0.89)	1.33 (1.10-1.62)
Behenic acid (22:0)	0.9 (0.7-1.2)	25.0 (20.8-31.0)	1.9 (1.5-2.3)	1.4 (1.1-1.6)	2.5 (2.0-3.0)
Lignoceric acid (24:0)	1.8 (1.2-2.4)	51.0 (41.0-65.0)	4.3 (3.4-5.7)	1.3 (1.2-1.5)	4.8 (3.4-5.6)

Abbreviations: FA = fatty acids, IQR = interquartile range (25th percentile to 75th percentile), MUFAs = monounsaturated fatty acids, PUFAs = polyunsaturated fatty acids, SFAs = saturated fatty acids.

Supplementary Table 2. Effect of post-mortem interval on association between blood and trigeminovascular tissue fatty acids

	Basilar Arteries, n=63				Meninges (Dura), n=64				Trigeminal Ganglia, n=69			
	Main Effect of Blood FA		Blood FA × PMI		Main Effect of Blood FA		Blood FA × PMI		Main Effect of Blood FA		Blood FA × PMI	
	Coefficient (95% CI)	P-value	Coefficient (95% CI)	P-value	Coefficient (95% CI)	P-value	Coefficient (95% CI)	P-value	Coefficient (95% CI)	P-value	Coefficient (95% CI)	P-value
Omega n-6												
LA	0.53 (-0.12, 1.18)	0.106	-0.009 (-0.06, 0.04)	0.695	0.48 (0.25, 0.70)	**<0.001**	0.005 (-0.01, 0.02)	0.578	0.28 (-0.19, 0.74)	0.241	0.016 (-0.02, 0.05)	0.410
GLA	-0.02 (-0.49, 0.46)	0.936	-0.009 (-0.05, 0.03)	0.671	0.27 (0.08, 0.46)	**0.007**	0.003 (-0.01, 0.02)	0.751	-0.11 (-0.32, 0.10)	0.303	-0.001 (-0.02, 0.02)	0.925
DGLA	0.25 (0.05, 0.46)	**0.015**	-0.009 (-0.03, 0.01)	0.320	0.17 (-0.07, 0.40)	0.156	0.005 (-0.02, 0.03)	0.594	0.31 (0.13, 0.49)	**0.001**	0.004 (-0.01, 0.02)	0.635
AA	0.08 (-0.31, 0.47)	0.680	-0.002 (-0.04, 0.03)	0.905	0.09 (-0.17, 0.35)	0.486	-0.007 (-0.03, 0.02)	0.547	0.22 (0.03, 0.41)	**0.026**	0.018 (-0.00, 0.04)	0.059
DTA	-0.11 (-0.44, 0.21)	0.478	0.004 (-0.02, 0.03)	0.758	-0.07 (-0.33, 0.20)	0.611	-0.007 (-0.03, 0.01)	0.482	0.16 (0.01, 0.32)	**0.039**	-0.001 (-0.01, 0.01)	0.818
DPA n-6	0.11 (-0.22, 0.43)	0.516	0.010 (-0.02, 0.04)	0.515	0.37 (0.14, 0.60)	**0.002**	-0.008 (-0.03, 0.01)	0.447	0.51 (0.34, 0.68)	**<0.001**	-0.004 (-0.02, 0.01)	0.652
Eicosadienoic acid	0.72 (0.24, 1.19)	**0.004**	-0.045 (-0.08, -0.01)	**0.009**	0.38 (0.12, 0.63)	**0.004**	-0.013 (-0.03, 0.00)	0.123	0.08 (-0.20, 0.37)	0.562	-0.025 (-0.05, -0.00)	**0.019**
Omega n-3												
ALA	0.29 (-0.15, 0.74)	0.194	0.016 (-0.03, 0.06)	0.454	0.56 (0.30, 0.82)	**<0.001**	0.028 (0.003, 0.053)	**0.028**	0.32 (-0.02, 0.66)	0.065	0.022 (-0.01, 0.05)	0.166
EPA	0.45 (0.05, 0.85)	**0.029**	0.036 (-0.02, 0.10)	0.224	0.39 (0.21, 0.56)	**<0.001**	0.008 (-0.01, 0.03)	0.392	0.22 (0.01, 0.42)	**0.041**	0.009 (-0.01, 0.03)	0.463
DPA n-3	-0.09 (-0.33, 0.16)	0.488	0.009 (-0.02, 0.03)	0.497	0.31 (0.10, 0.52)	**0.004**	-0.024 (-0.046, -0.003)	**0.029**	0.29 (0.16, 0.41)	**<0.001**	0.001 (-0.01, 0.01)	0.837
DHA	0.00 (-0.54, 0.55)	0.987	0.013 (-0.05, 0.07)	0.660	0.01 (-0.35, 0.38)	0.937	0.036 (-0.00, 0.07)	0.068	0.31 (0.13, 0.48)	**0.001**	0.004 (-0.01, 0.02)	0.650
MUFAs												
Palmitoleic acid	0.24 (0.09, 0.38)	**0.003**	0.004 (-0.01, 0.02)	0.591	0.48 (0.29, 0.68)	**<0.001**	0.013 (-0.00, 0.03)	0.148	0.23 (0.07, 0.38)	**0.005**	0.004 (-0.01, 0.02)	0.604
Oleic acid	0.31 (0.04, 0.57)	**0.024**	0.021 (0.00, 0.04)	**0.038**	0.28 (0.09, 0.47)	**0.006**	0.009 (-0.01, 0.02)	0.256	0.16 (0.04, 0.27)	**0.007**	0.006 (-0.00, 0.02)	0.217
Cis-vaccenic acid	0.10 (-0.13, 0.33)	0.389	0.009 (-0.01, 0.03)	0.331	0.32 (0.19, 0.45)	**<0.001**	0.020 (0.01, 0.03)	**0.001**	0.21 (0.10, 0.32)	**<0.001**	0.005 (-0.00, 0.01)	0.262
Eicosenoic acid	0.67 (-0.13, 1.47)	0.100	-0.006 (-0.07, 0.06)	0.853	0.27 (0.07, 0.47)	**0.009**	-0.002 (-0.02, 0.01)	0.798	-0.06 (-0.25, 0.13)	0.558	-0.011 (-0.03, 0.00)	0.157
Erucic acid	0.03 (-0.28, 0.34)	0.867	-0.004 (-0.04, 0.04)	0.847	0.18 (-0.15, 0.50)	0.274	0.023 (-0.02, 0.06)	0.266	0.00 (-0.17, 0.17)	0.964	0.006 (-0.01, 0.02)	0.490
Nervonic acid	0.28 (-0.04, 0.59)	0.088	-0.008 (-0.03, 0.02)	0.548	0.09 (-0.08, 0.27)	0.293	0.005 (-0.01, 0.02)	0.504	0.24 (0.05, 0.44)	**0.017**	0.012 (-0.00, 0.03)	0.142
SFAs												
Lauric acid					0.26 (0.13, 0.39)	**<0.001**	-0.004 (-0.02, 0.02)	0.666				
Myristic acid	0.23 (0.06, 0.40)	**0.008**	-0.007 (-0.02, 0.01)	0.436	0.34 (0.18, 0.49)	**<0.001**	-0.007 (-0.02, 0.01)	0.380	0.12 (-0.05, 0.28)	0.165	-0.002 (-0.02, 0.01)	0.812
Palmitic acid	-0.11 (-0.50, 0.28)	0.572	0.017 (-0.02, 0.05)	0.360	0.18 (-0.03, 0.39)	0.083	0.004 (-0.02, 0.02)	0.726	0.16 (-0.12, 0.44)	0.261	-0.003 (-0.03, 0.02)	0.855
Stearic acid	-0.08 (-0.41, 0.25)	0.634	-0.002 (-0.03, 0.03)	0.904	-0.15 (-0.42, 0.12)	0.254	0.027 (0.00, 0.05)	**0.025**	0.87 (-0.17, 1.91)	0.097	-0.006 (-0.09, 0.08)	0.890
Arachidic acid (20:0)	0.22 (-0.12, 0.56)	0.199	0.011 (-0.02, 0.04)	0.422	0.14 (-0.11, 0.39)	0.271	0.009 (-0.01, 0.03)	0.361	0.20 (-0.10, 0.49)	0.188	0.022 (-0.00, 0.05)	0.059
Behenic acid (22:0)	0.16 (-0.14, 0.45)	0.284	0.002 (-0.02, 0.02)	0.869	0.11 (-0.09, 0.32)	0.277	0.008 (-0.01, 0.02)	0.345	0.25 (0.02, 0.48)	**0.033**	0.016 (-0.00, 0.03)	0.093
Lignoceric acid (24:0)	0.18 (-0.08, 0.45)	0.174	0.011 (-0.01, 0.03)	0.267	0.07 (-0.06, 0.21)	0.285	0.003 (-0.01, 0.01)	0.582	0.15 (-0.04, 0.34)	0.120	0.009 (-0.01, 0.02)	0.239

1. Regression models were fit separately by tissue with fatty acids (percent of total) log-transformed to stabilize variance and improve fit. "Main effect of blood FA" is the percent change in tissue FA associated with a 1% increase in blood FA—evaluated at the median PMI for the model's estimation sample. "Blood FA × PMI" indicates how that association changes per one-hour increase in PMI. Models adjust for PMI, blood FA×PMI, headache status, age, body mass index, sex, race, manner of death, geographic source, and positive ethyl alcohol test.
2. P-values in bold are less than 0.05.
Abbreviations: FA = fatty acid, MUFAs = monounsaturated fatty acids, PMI = post-mortem interval, PUFAs = polyunsaturated fatty acids, SFAs = saturated fatty acids.

Supplementary Table 3. Percent of total fatty acids by headache group

	Blood, n=70		Basilar Arteries, n=63		Meninges (Dura), n=64		Trigeminal Ganglia, n=69	
	Median (IQR)	P-value	Median (IQR)	P-value	Median (IQR)	P-value	Median (IQR)	P-value
Omega n-6								
LA								
No headache	20.13 (17.25-23.79)	0.40	3.90 (3.32-5.18)	0.09	10.41 (8.93-11.61)	0.48	6.04 (4.37-8.21)	0.83
Headache	18.97 (16.59-22.16)		3.09 (2.28-5.82)		10.09 (7.96-11.28)		5.41 (4.22-8.89)	
GLA								
No headache	0.19 (0.14-0.27)	0.59	0.06 (0.04-0.09)	0.49	0.09 (0.06-0.10)	0.45	0.03 (0.02-0.04)	0.86
Headache	0.20 (0.11-0.33)		0.06 (0.04-0.11)		0.09 (0.07-0.11)		0.03 (0.02-0.04)	
DGLA								
No headache	1.03 (0.74-1.26)	0.07	1.02 (0.90-1.14)	0.84	0.91 (0.67-1.12)	0.74	1.07 (0.85-1.24)	0.46
Headache	1.21 (0.98-1.46)		0.97 (0.85-1.32)		0.91 (0.69-0.98)		1.18 (0.86-1.31)	
AA								
No headache	6.09 (4.86-7.17)	0.33	8.30 (6.34-9.83)	0.65	4.83 (3.70-6.03)	0.49	4.17 (3.62-4.98)	0.90
Headache	6.66 (4.87-8.25)		8.99 (7.16-9.91)		4.37 (3.68-5.83)		4.35 (3.50-4.75)	
DTA								
No headache	0.86 (0.64-1.21)	0.81	1.80 (1.14-2.19)	0.28	1.22 (0.86-1.65)	0.39	3.05 (2.39-3.57)	0.76
Headache	0.84 (0.69-1.03)		1.94 (1.53-2.50)		1.06 (0.83-1.51)		3.03 (2.29-3.54)	
DPA n-6								
No headache	0.18 (0.15-0.25)	0.21	0.59 (0.50-0.80)	0.31	0.27 (0.22-0.32)	0.14	0.24 (0.19-0.29)	0.88
Headache	0.22 (0.16-0.25)		0.67 (0.50-0.99)		0.23 (0.16-0.31)		0.24 (0.18-0.31)	
Eicosadienoic acid								
No headache	0.29 (0.26-0.34)	0.23	0.31 (0.26-0.40)	0.20	0.36 (0.31-0.44)	0.09	1.06 (0.84-1.23)	0.05
Headache	0.28 (0.23-0.32)		0.29 (0.25-0.33)		0.33 (0.29-0.39)		0.90 (0.79-1.10)	
Omega n-3								
ALA								
No headache	0.53 (0.41-0.75)	0.30	0.15 (0.08-0.23)	0.73	0.17 (0.14-0.23)	0.31	0.15 (0.07-0.28)	0.99
Headache	0.47 (0.41-0.60)		0.14 (0.08-0.29)		0.15 (0.11-0.25)		0.12 (0.10-0.32)	
EPA								
No headache	0.23 (0.16-0.29)	0.39	0.08 (0.06-0.11)	0.60	0.07 (0.06-0.09)	0.57	0.06 (0.05-0.08)	0.84
Headache	0.27 (0.18-0.34)		0.09 (0.07-0.11)		0.07 (0.06-0.11)		0.06 (0.05-0.08)	

	Blood, n=70		Basilar Arteries, n=63		Meninges (Dura), n=64		Trigeminal Ganglia, n=69	
	**Median (IQR)**	**P-value**	**Median (IQR)**	**P-value**	**Median (IQR)**	**P-value**	**Median (IQR)**	**P-value**
DPA n-3								
No headache	0.57 (0.42-0.72)	0.95	0.41 (0.27-0.49)	1.00	0.30 (0.22-0.41)	0.55	0.69 (0.53-0.81)	1.00
Headache	0.55 (0.39-0.73)		0.39 (0.31-0.50)		0.28 (0.20-0.37)		0.66 (0.56-0.80)	
DHA								
No headache	0.81 (0.60-1.17)	0.63	1.63 (0.95-2.52)	0.66	0.63 (0.36-0.96)	0.61	0.78 (0.61-0.99)	0.58
Headache	0.91 (0.63-1.21)		1.71 (1.16-2.43)		0.52 (0.34-0.90)		0.74 (0.62-0.93)	
MUFAs								
Palmitoleic acid								
No headache	1.74 (1.20-2.44)	0.61	0.60 (0.44-0.84)	0.84	0.96 (0.71-1.61)	0.70	0.92 (0.64-1.23)	0.33
Headache	1.71 (1.21-3.02)		0.61 (0.48-0.80)		1.11 (0.78-1.69)		0.92 (0.71-1.36)	
Oleic acid								
No headache	20.1 (18.3-23.3)	0.41	18.2 (14.8-20.5)	0.85	20.3 (19.3-23.7)	0.14	30.7 (29.3-32.3)	0.33
Headache	21.2 (18.5-24.9)		17.7 (16.0-18.9)		21.8 (20.1-23.8)		31.4 (29.4-33.8)	
Cis-vaccenic acid								
No headache	1.86 (1.68-2.12)	0.65	3.05 (2.30-3.60)	0.76	2.09 (1.84-2.36)	0.58	2.75 (2.48-3.03)	0.47
Headache	1.90 (1.66-2.25)		2.91 (2.42-3.58)		1.99 (1.79-2.28)		2.70 (2.45-2.85)	
Eicosenoic acid								
No headache	0.24 (0.20-0.28)	0.83	0.84 (0.38-1.39)	0.97	0.46 (0.41-0.54)	0.61	2.93 (2.44-3.49)	0.51
Headache	0.23 (0.19-0.30)		0.67 (0.40-1.24)		0.49 (0.42-0.56)		2.92 (2.50-3.20)	
Erucic acid								
No headache	0.06 (0.05-0.07)	**0.97**	0.34 (0.25-0.45)	0.03	0.51 (0.38-0.60)	0.51	0.50 (0.43-0.60)	0.87
Headache	0.06 (0.04-0.08)		0.27 (0.19-0.37)		0.48 (0.36-0.78)		0.51 (0.44-0.56)	
Nervonic acid								
No headache	1.62 (1.32-2.19)	0.82	2.72 (2.19-3.41)	0.33	1.76 (1.55-2.03)	0.84	5.06 (3.54-5.87)	0.51
Headache	1.68 (1.14-2.38)		2.34 (2.01-3.36)		1.70 (1.42-2.05)		4.75 (3.61-5.48)	
SFAs								
Lauric acid								
No headache	0.15 (0.08-0.19)	0.50			0.16 (0.11-0.22)	0.65		.
Headache	0.12 (0.07-0.20)				0.18 (0.13-0.23)			

	Blood, n=70		Basilar Arteries, n=63		Meninges (Dura), n=64		Trigeminal Ganglia, n=69	
	**Median (IQR)**	**P-value**	**Median (IQR)**	**P-value**	**Median (IQR)**	**P-value**	**Median (IQR)**	**P-value**
Myristic acid								
No headache	1.17 (0.96-1.40)	0.81	1.04 (0.94-1.15)	0.28	0.93 (0.82-1.18)	0.27	1.47 (1.27-1.69)	0.54
Headache	1.17 (0.74-1.61)		1.00 (0.86-1.17)		1.00 (0.87-1.23)		1.59 (1.27-1.92)	
Palmitic acid								
No headache	26.8 (24.8-28.3)	0.75	25.8 (22.9-27.5)	0.97	31.0 (29.3-33.2)	0.75	19.1 (16.9-20.6)	0.98
Headache	26.8 (25.2-27.4)		25.8 (23.4-27.1)		31.1 (28.8-32.8)		18.7 (17.1-20.9)	
Stearic acid								
No headache	9.67 (8.46-11.08)	0.33	20.69 (18.52-22.34)	0.32	17.19 (15.59-18.98)	0.89	10.14 (9.26-11.03)	0.85
Headache	9.31 (8.41-10.25)		21.52 (20.26-23.16)		17.73 (15.34-18.93)		10.25 (9.15-11.48)	
Arachidic acid (20:0)								
No headache	0.29 (0.26-0.35)	0.79	0.78 (0.60-0.89)	0.08	0.75 (0.68-0.84)	0.65	1.33 (1.14-1.65)	0.77
Headache	0.33 (0.26-0.37)		0.66 (0.57-0.80)		0.78 (0.66-0.92)		1.38 (1.07-1.62)	
Behenic acid (22:0)								
No headache	0.88 (0.69-1.11)	0.76	2.01 (1.60-2.48)	0.09	1.33 (1.07-1.53)	0.36	2.46 (2.00-2.96)	0.95
Headache	0.85 (0.67-1.15)		1.64 (1.33-2.15)		1.45 (1.15-1.68)		2.54 (1.85-3.01)	
Lignoceric acid (24:0)								
No headache	1.78 (1.21-2.34)	0.48	4.28 (3.40-5.98)	0.73	1.30 (1.10-1.49)	0.55	4.65 (3.39-5.57)	0.88
Headache	1.69 (1.03-2.37)		4.26 (3.02-5.58)		1.36 (1.17-1.53)		4.68 (3.48-5.62)	

P-values based on Wilcoxon rank-sum tests.
Abbreviations: IQR = interquartile range (25th percentile to 75th percentile).

Supplementary Table 4. Difference in percent of total fatty acids between headache status groups

	Blood, n=64		Basilar Arteries, n=57		Meninges (Dura), n=58		Trigeminal Ganglia, n=63	
	Coefficient (95% CI)	P-value	Coefficient (95% CI)	P-value	Coefficient (95% CI)	P-value	Coefficient (95% CI)	P-value
Omega n-6								
LA	-0.01 (-0.16, 0.13)	0.838	-0.18 (-0.50, 0.14)	0.264	-0.03 (-0.15, 0.10)	0.691	-0.04 (-0.28, 0.19)	0.715
GLA	-0.17 (-0.53, 0.19)	0.350	0.23 (-0.34, 0.79)	0.427	0.08 (-0.15, 0.32)	0.484	-0.11 (-0.38, 0.15)	0.391
DGLA	-0.05 (-0.27, 0.18)	0.674	0.02 (-0.15, 0.19)	0.795	0.03 (-0.14, 0.20)	0.732	0.03 (-0.12, 0.19)	0.665
AA	-0.08 (-0.30, 0.13)	0.445	-0.04 (-0.31, 0.23)	0.786	0.03 (-0.14, 0.20)	0.695	0.04 (-0.12, 0.20)	0.625
DTA	-0.10 (-0.40, 0.20)	0.490	0.05 (-0.24, 0.34)	0.740	-0.04 (-0.28, 0.20)	0.751	0.02 (-0.13, 0.16)	0.827
DPA n-6	0.05 (-0.19, 0.29)	0.674	0.06 (-0.22, 0.34)	0.657	-0.12 (-0.34, 0.09)	0.252	0.04 (-0.15, 0.23)	0.665
Eicosadienoic acid	-0.05 (-0.19, 0.08)	0.453	-0.05 (-0.28, 0.19)	0.697	-0.03 (-0.14, 0.08)	0.603	-0.07 (-0.20, 0.07)	0.317
Omega n-3								
ALA	-0.04 (-0.33, 0.25)	0.785	0.14 (-0.30, 0.57)	0.534	-0.11 (-0.42, 0.21)	0.495	-0.05 (-0.43, 0.32)	0.769
EPA	-0.28 (-0.61, 0.06)	0.100	0.03 (-0.41, 0.47)	0.879	0.06 (-0.17, 0.29)	0.612	-0.17 (-0.41, 0.08)	0.173
DPA n-3	-0.21 (-0.50, 0.08)	0.159	-0.05 (-0.29, 0.19)	0.652	-0.01 (-0.22, 0.21)	0.949	0.00 (-0.15, 0.15)	0.983
DHA	-0.04 (-0.32, 0.23)	0.749	0.02 (-0.49, 0.52)	0.952	0.02 (-0.31, 0.36)	0.889	0.04 (-0.14, 0.23)	0.630
MUFAs								
Palmitoleic acid	-0.15 (-0.55, 0.26)	0.472	-0.13 (-0.38, 0.11)	0.281	-0.14 (-0.50, 0.23)	0.453	0.01 (-0.23, 0.25)	0.929
Oleic acid	0.04 (-0.06, 0.15)	0.405	-0.03 (-0.13, 0.07)	0.603	-0.03 (-0.10, 0.05)	0.487	0.01 (-0.04, 0.05)	0.759
Cis-vaccenic acid	0.05 (-0.12, 0.22)	0.562	-0.04 (-0.18, 0.10)	0.573	-0.04 (-0.14, 0.06)	0.423	-0.03 (-0.10, 0.04)	0.453
Eicosenoic acid	0.02 (-0.15, 0.19)	0.818	0.03 (-0.44, 0.50)	0.892	0.00 (-0.12, 0.12)	0.964	-0.07 (-0.18, 0.05)	0.249
Erucic acid	-0.01 (-0.24, 0.21)	0.906	-0.13 (-0.37, 0.12)	0.294	0.12 (-0.13, 0.37)	0.342	-0.02 (-0.16, 0.11)	0.740
Nervonic acid	-0.09 (-0.34, 0.16)	0.479	0.03 (-0.24, 0.29)	0.839	0.07 (-0.08, 0.22)	0.344	-0.01 (-0.20, 0.18)	0.948
SFAs								
Lauric acid	-0.35 (-0.81, 0.11)	0.133			-0.08 (-0.33, 0.17)	0.507		
Myristic acid	-0.07 (-0.32, 0.19)	0.601	-0.06 (-0.22, 0.10)	0.450	-0.05 (-0.21, 0.10)	0.483	0.01 (-0.14, 0.16)	0.921
Palmitic acid	-0.02 (-0.10, 0.05)	0.524	-0.01 (-0.09, 0.07)	0.817	0.01 (-0.04, 0.06)	0.662	-0.02 (-0.09, 0.04)	0.458
Stearic acid	-0.04 (-0.13, 0.05)	0.373	0.02 (-0.07, 0.11)	0.660	0.02 (-0.05, 0.10)	0.523	-0.05 (-0.37, 0.27)	0.765
Arachidic acid (20:0)	-0.01 (-0.15, 0.14)	0.934	-0.07 (-0.25, 0.11)	0.445	0.06 (-0.07, 0.19)	0.375	0.01 (-0.15, 0.16)	0.910
Behenic acid (22:0)	-0.07 (-0.27, 0.13)	0.496	-0.06 (-0.26, 0.15)	0.580	0.07 (-0.08, 0.22)	0.344	0.02 (-0.16, 0.20)	0.820
Lignoceric acid (24:0)	-0.12 (-0.41, 0.17)	0.410	0.05 (-0.21, 0.32)	0.693	0.06 (-0.06, 0.19)	0.319	0.03 (-0.16, 0.23)	0.732

For each tissue, regressions were conducted on log-transformed fatty acid levels versus headache status and adjusted for age, body mass index, sex, race, manner of death, postmortem interval, geographic source, and positive ethyl alcohol test. Differences in sample sizes are due to six cases missing confounders and a few other cases missing tissues.
Abbreviations: CI = confidence interval.
